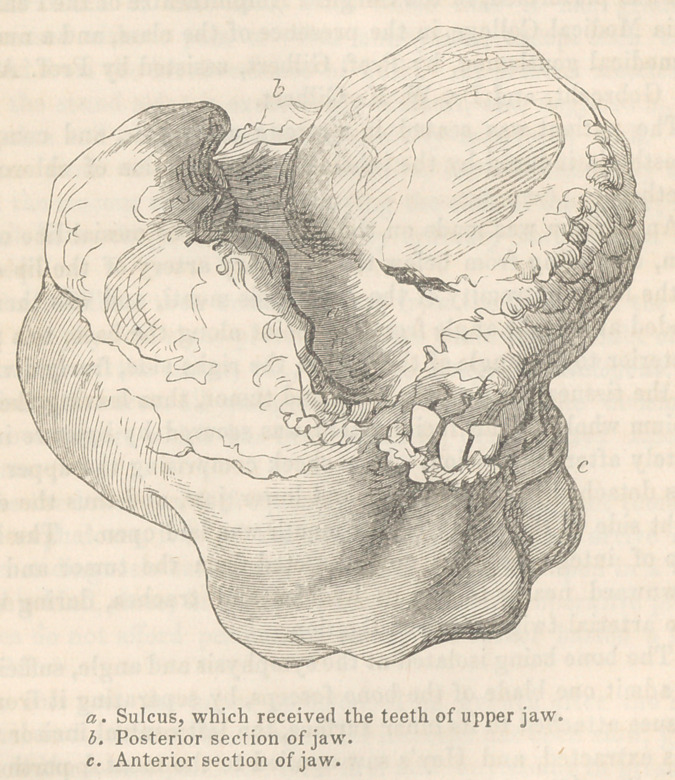# Pennsylvania College, Ninth below Locust Street

**Published:** 1853-12

**Authors:** W. H. Gobrecht


### CLINICAL REPORTS.

*Pennsylvania College, Ninth below Locust street. Service of* Professor Gilbert.

Reported by W. H. Gobrecht, M. D.

*Case of Extensive Enchondroma of the inferior maxilla, removed by resection of the body of the bone from the symphysis to the angle on the right side.*

The patient, Moses Christopher, aged thirty years, colored, seaman by occupation, generally residing in this city and enjoying good health, was brought before the class by Professor Gilbert on Oct. 12th, 1853, when it was shown that a large tumor projected from the right side of his face, commencing immediately below the malar bone and extending into the neck, whilst horizontally it occupied all the space intervening between the symphysis of the chin and the lobe of the ear. The outlines of the tumour were distinct, terminating abruptly, without adhesion to the integument or any tissue or organ bounding it. The cavity of the mouth was found filled by the tumor which was traversed by a deep sulcus that received the row of teeth projecting from the right superior maxilla, around which the tumor seemed to have moulded itself. Of the two lobes thus formed, one extending upward and outward greatly distended the cheek, and the other passing upward and inward almost completely filled the whole palatine arch of the mouth when it was closed. The tongue was pushed back, so as to be seen with difficulty, and the os hyoides and larynx were also pressed toward the pharynx by the extension of the tumor in that direction.

The external appearance of the tumor is here shown in a cut by Gihon, from a Daguerreotype by Laughlin.

This entire adventitious mass seemed to consist of an expansion of, or an interstitial growth within, the right half of the inferior maxilla, terminating abruptly at the symphysis and angle of the bone. It is hard and osseous in all its periphery; within the mouth, it is covered by the natural tissues which remain free from abrasion or ulceration. The lower teeth of this side are covered by and involved in the tumor.

Of the numerous cases of tumor operated for already in the presence of the class this session, no one belongs to the division of which this is a most interesting specimen.

The patient stated that he observed the commencement of this tumor, about four years ago, he then had pain in a molar tooth of that side, since which time it has gradually increased without pain or other annoyance, except such as resulted from the inconvenience of bulk. At present mastication (on the sound side) is exceedingly difficult, and deglutition, respiration and articulation, are materially interfered with. The molar teeth of the superior maxilla, have occasionally abraded the mucous membrane occupying the cleft of the tumor, from which hemorrhage occurred, but by ordinary care it soon cicatrized.

Prof. Gilbert then remarked that from the history of the case and the physical signs presented, there could be no doubt of the character of the tumor, viz., that it is *benign* or *analogous*, and entirely free from all malignancy, belonging to the division of osseous tumors which has been recently denominated Enchondroma.

As the lower jaw is frequently the seat of malignant tumor or osteocephaloma, it is of the utmost importance to arrive at a correct diagnosis before any operation is decided upon in a case of this kind, since, in the latter form of disease, operative procedures do not afford permanent relief, but rather hasten a fatal result.

In this case the tumor commenced its growth after the individual had attained adult age; its increase has been slow, gradual, uniform and painless; it is firm and unyielding to pressure, and presents an even convex surface; it has formed no attachment with surrounding tissues, nor do we observe any disorder of the general system; there are no ulcerating points upon its surface, all abrasion from the action of the upper teeth having cicatrized. Dr. Gilbert has therefore no hesitation in deciding upon the propriety of an operation, and will expect it to be quite as successful as that performed here last year, in the case of Mr. Reilly, involving the removal of a smaller portion of the lower jaw, or that of Mrs. E. McB——, of N. J., in whose case about a year ago the entire superior maxilla was removed, both precisely similar in their histological characters. The operation though formidable and involving risk to life, is nevertheless justified by the impending fatal result if left to itself, by mechanical closure of the larynx and œsophagus.

*On Wednesday, Oct.* 19*th*, at 1 P. M., the operation of resection was performed, in the Surgical Amphitheatre of the Pennsylvania Medical College, in the presence of the class, and a number of medical gentlemen, by Prof. Gilbert, assisted by Prof. Allen, Dr. Gobrecht, and Dr. W. K. Gilbert.

The patient was seated in a strong arm chair, and complete anæsthesia induced by the inhalation of a solution of chloroform in ether.

An incision was made on the left side of the mesial line of the chin, extending from below the coronary artery of the lip down to the lower extremity of the symphysis menti, and was then extended at a right angle from this point along the base, to a point posterior to the angle of the jaw on the right side, freely dividing all the tissues down to the bone and tumor, thus leaving the prolabium whole. The facial artery was secured by ligature immediately after its division. The cheek comprising the upper flap, was detached from the tumor and lower jaw, and thus the entire right side of the cavity of the mouth was laid open. The lower flap of integument was now dissected from the tumor and jaw, downward nearly to the os hyoides and trachea, during which two arterial twigs required ligation.

The bone being isolated at the symphysis and angle, sufficiently to admit one blade of the bone forceps, by separating it from the tissues attached to its inner surface, the left central incisor tooth was extracted, and Hey's saw applied to the mental portion externally, obliquely from the left side of the median line above to the right side below, so as to preserve the genial tubercles within, to which the anterior muscles of the tongue and the elevators of the os hyoides are attached, and thus obviate the necessity of transfixing the tongue by tenaculum or ligature to prevent suffocation from its retraction. The saw was next applied to the angle, and the division of the osseous tissue at both points completed by the bone-forceps. The tumor was then separated from its remaining attachments by dissection within the mouth, where two more ligatures were applied to branches of the lingual artery, and the actual cautery employed at one or two points, which completely arrested the hæmorrhage.

On examination of the tumor and the chasm made by its removal, it was found that the adventitious mass—as here shown in a cut by Gihon, from a drawing by Dr. Jeremy Wilson—was en-

*a*. Sulcus, which received the teeth of upper jaw.

*b*. Posterior section of jaw.

*c*. Anterior section of jaw.

tirely abstracted, and that the surrounding tissues and organs had merely been pushed aside by it. The submaxillary and sublingual glands, as well as the muscles of the locality, were distinctly in view, although displaced, by the previous pressure of the tumor.

The bone at the points of section was perfectly healthy, although unusually compact and hard, which somewhat embarrassed its resection.

The patient lost about twelve ounces of blood, but the pulse did not flag under the operation.

A silver plate, previously moulded to the teeth of the superior and inferior maxillæ of the left side by Dr. Ingram, was now applied, and the remaining portion of the lower jaw thus fixed in its proper position, when the wound was closed by the introduction of seven needles forming twisted sutures, adhesive strips being placed between. The space formerly occupied by the tumor was partly filled with dossils of lint, which restored the symmetry of the face, and a narrow bandage applied beneath the jaw and over the vertex prevented any displacement of the parts. Cold water dressings were ordered.

The patient was carried to his bed in an adjoining room; the pulse being 100 in the minute.

In the evening, pulse was 108; patient comfortable.

At 10 o'clock, P. M., pulse 120. Sol. morphiæ sulphat. fʒij administered.

*Oct. 20th*, 9 o'clock, A. M.—Has slept well; pulse 108, full and soft. Complains of no pain.

21*st.*—Pulse 98. Renewed the lint, and administered magnesiæ sulphat. ℥j, which operated twice.

22*d*, 1 o'clock, P. M.—Exhibited patient to the class. Progressing favorably; the external wound has united throughout its entire extent, except where the arterial ligatures pass out. Needles removed and adhesive plasters renewed.

23*d.*—Again renewed the lint. Pulse 90, and all other symptoms favorable.

From this time forward the patient recovered rapidly.

The tumor was examined, and found to be homologous in structure, being an Enchondroma. The elementary constituents, as proven by the microscope, are benign in their character.

The patient was exhibited to the class on Saturday, Nov. 19th, and discharged cured. The line of incision being for the most part under the base of the face, can hardly be observed. The vertical section on the chin, not having extended through the prolabium, leaves but a trifling cicatrix. The contour of the face is not materially affected, and its symmetry very slightly interfered with.

## Figures and Tables

**Figure f1:**
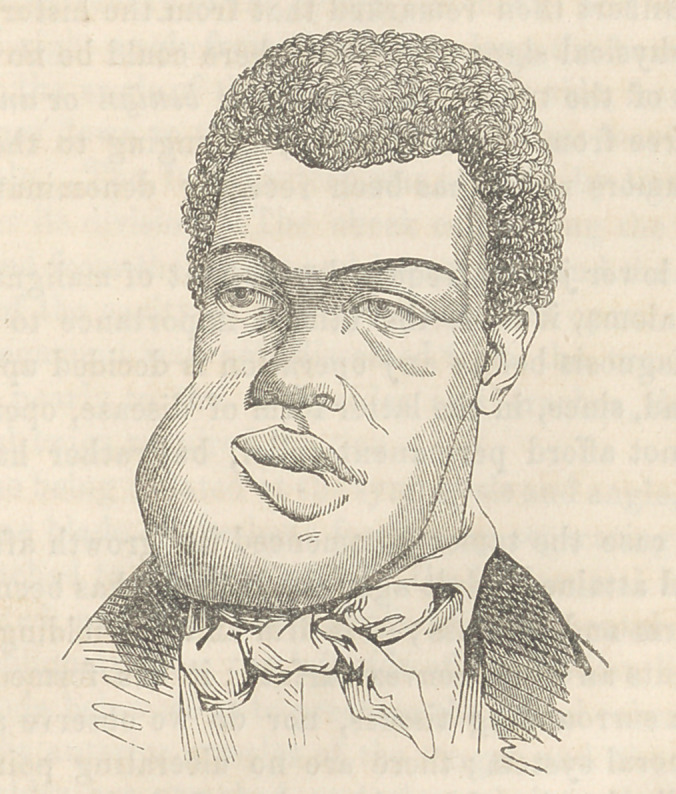


**Figure f2:**